# Exploring the relationship between mindfulness and burnout among preschool teachers: the role of dispositional equanimity and empathy

**DOI:** 10.3389/fpsyg.2024.1312463

**Published:** 2024-06-04

**Authors:** Yuanqing He, Xin Wang, Xiaqing Wang, Xiaowen Li

**Affiliations:** ^1^School of Educational Science, AnHui Normal University, Wuhu, China; ^2^School of Humanities and Social, Chosun University, Gwangju, Republic of Korea

**Keywords:** burnout, dispositional equanimity, empathy, mindfulness, preschool teachers

## Abstract

The objective of this study is to delve into the underlying mechanisms between mindfulness and burnout among preschool teachers. Employing a cross-sectional research design, this study surveyed 1,980 Chinese preschool teachers using the Mindful Attention Awareness Scale (MAAS), Preschool Teacher Dispositional Equanimity Questionnaire (PTDEQ), Empathy Scale (ME), and Maslach Burnout Inventory for Educators (MBI-ES). The results revealed a significant negative correlation between preschool teachers’ mindfulness and burnout. A mediation analysis demonstrated that dispositional equanimity served as a mediator between mindfulness and preschool teacher burnout. Furthermore, a moderation analysis indicated that empathy moderated the influence of dispositional equanimity on preschool teacher burnout. These findings suggest that mindfulness can enable preschool teachers to better cope with workplace challenges with a more peaceful mindset.

## 1 Introduction

The rising national demand for advanced early childhood education has exacerbated the pressures and challenges faced by educators in this sector. Coupled with the special task of integrating care and education, the risk of experiencing professional burnout has been increasing. Professional burnout is better understood as an occupational issue rather than an individualized concern. In China, the prevalence of occupational burnout among early childhood educators is notably elevated. This is due to their long-term engagement in demanding and high-pressure jobs, making them a group worthy of special attention (Li et al., 2020). Preschool teachers are educators for children aged 3–6. Compared to teachers in other grade levels, they not only receive less salary and respect, but also face a relatively unique professional ecological environment. Apart from kindergarten teachers who are partly civil servants, many preschool teachers in China are on temporary service contracts. Most of them are under contract employment, characterized by high temporality and uncertainty, with no guarantee of lifelong employment. Especially since the outbreak of the pandemic, the early childhood education industry has been greatly affected. During the pandemic, many kindergartens struggled to operate, leading to a loss of income for numerous preschool teachers. This resulted in increased fatigue and a more negative perception and emotional attitude toward their work and profession (Wei et al., 2021). Additionally, the sudden outbreak of the pandemic significantly increased teachers’ cognitive and emotional burdens. The shortage of teaching and research resources caused by the pandemic further intensifies the pressure and fatigue experienced by preschool teachers (Sokal et al., 2020). Preschool teachers burnout not only affects the physical and mental health and quality of life of early childhood educators but also increases the risk of harming and seriously abusing children. For a long time, early childhood educators have had low social status, low wages, and most are employed on contract basis, which brings about temporary and uncertain employment, making this group of educators less resilient to risk. During the pandemic, many kindergartens struggled to operate, leading to a loss of income for numerous preschool teachers. This resulted in increased fatigue and a more negative perception and emotional attitude toward their work and profession (Wei et al., 2021). Additionally, the sudden outbreak of the pandemic significantly increased teachers’ cognitive and emotional burdens. The shortage of teaching and research resources caused by the pandemic further intensifies the pressure and fatigue experienced by preschool teachers (Sokal et al., 2020).

Preschool teachers burnout refers to a series of sustained negative reactions and behaviors that occur when teachers are in a state of long-term high workloads, long hours, and high intensity. Specifically, it manifests specifically in emotional exhaustion, depersonalization, and reduced personal accomplishment (Maslach et al., 2001). The Job Demands-Resources (JD-R) model, serves as a pivotal theoretical framework for understanding occupational burnout. The model suggests that the characteristics of any job can be divided into job demands and job resources. Job demands are the “negative factors” that consume an individual’s energy in the workplace, while job resources are the “positive factors” in the work environment (Demerouti et al., 2001). Job demands are positively correlated with burnout, while job resources are often negatively correlated with burnout (Jeon et al., 2022). Numerous empirical studies have explored the influencing factors of burnout among early childhood educators from organizational and environmental perspectives, such as the person-organization fit (Zang and Chen, 2022), work stress (Guo et al., 2022), and organizational climate (Ji and Yue, 2020). However, only a handful of studies have examined the individual factors that influence preschool teacher burnout. For instance, a randomized controlled trial explored the impact of preschool teachers’ self-efficacy on burnout (Roberts et al., 2020). Additionally, a cross-sectional study elucidated the relationship between preschool teachers’ emotional labor strategies, psychological capital, and burnout (Peng et al., 2019). The concept of mindfulness is classically esteemed, and practices centered around it have been confirmed to safeguard individuals from the pernicious effects of burnout among educators. Nevertheless, the underlying mechanisms explaining why mindfulness can mitigate burnout remain elusive (Leroy et al., 2013). Prior research suggests that mindfulness fosters inner tranquility and stability (Michon and Rosemead, 2014), thereby facilitating individuals’ coping strategies in the face of emotional stress in both life and work (Pigni, 2014). Given the unique nature of preschool teachers, they are often confronted with numerous emotional demands in their work (Wang et al., 2019). Empathy, as a valuable work resource, can assist preschool teachers in better managing these emotional demands. This study aims to explore the influencing factors and mechanisms of burnout among early childhood educators from the perspective of their personal resources, in order to propose effective strategies for preventing professional burnout among early childhood educators.

### 1.1 Mindfulness and early childhood educator burnout

Within occupational health psychology, mindfulness is regarded as a personal trait or state (Dane and Brummel, 2014). Mindfulness involves an open, non-judgmental attitude wherein individuals consciously observe their physical and emotional states, focusing attentively on the present moment. Mindfulness serves not only as an internal psychological resource for early childhood educators but also as an important individual work resource (Zhang, 2020). Built upon the Job Demands-Resources model, mindfulness is conceptualized as a personal resource that can influence both job demands and job resources, interacting with them, and consequently impacting teacher burnout (Guidetti et al., 2019). Within the JD-R model, mindfulness serves as a versatile and potent resource with manifold benefits (Grover et al., 2017). A systematic review encompassing eight studies has shown that mindfulness is proven to be a positive characteristic for coping with burnout in numerous research: mindfulness practices can reduce job burnout among healthcare professionals and teachers (Luken and Sammons, 2016).

High levels of mindfulness can serve as personal work resources, enabling individuals to self-regulate, maintain emotional stability and a calm mindset to reduce emotional stress in the workplace (Grover et al., 2017).

### 1.2 The mediating role of dispositional equanimity

Equanimity is a personality disposition characterized by calmness, gentleness, and composure, which manifests as inner calmness, gentle interactions with others, and rationality in dealing with situations (He, 2020). Personality traits significantly contribute to burnout development, with specific traits shown to influence the prevalence of burnout among early childhood educators (Ghorpade et al., 2007; Tasic et al., 2020). High levels of neuroticism, signifying low emotional stability, are strongly correlated with increased burnout risk among teachers (Cramer and Binder, 2015). As a personality trait variable, dispositional equanimity can enhance emotional stability and influence individuals’ cognition and behavior (He, 2020). Literature confirms that if a person possesses positive personality traits, burnout is likely to be low (Khalil et al., 2017). Equanimity, as a positive personality disposition, may have a positive effect on alleviating preschool teachers burnout.

Mindfulness may reduce teacher burnout by enhancing early childhood educators’ dispositional equanimity. Inner calmness is one manifestation of dispositional equanimity in individuals, and previous research has found a significant positive correlation between mindfulness and inner calmness, indicating that mindfulness meditation can cultivate equanimity (Ren et al., 2012). The mindfulness coping model proposes that when an individual faces stressors that exceed their tolerance, they respond adaptively by assessing the stressors mindfully. This involves focusing on the dynamic process of consciousness rather than its content, thereby enhancing attention and cognitive flexibility. With this expanded metacognitive state, individuals actively reevaluate the stressors, reframe or reconstruct the stressful events. Ultimately, this process generates positive emotions, such as compassion, trust, self-assurance, or inner calm, which can alleviate the impact of stressors (Garland et al., 2009). Equanimity is manifested as a positive emotion in situations, and mindfulness can enhance positive emotions and alleviate negative emotions (Anderson et al., 2007). Studies on athletes and junior healthcare providers have found that positive and negative emotions mediate the relationship between mindfulness and burnout, suggesting that the relationship between burnout and mindfulness is partly regulated by positive and negative emotions (Gustafsson et al., 2015). Mindfulness-based interventions may not only directly impact the level of burnout but also indirectly affect it through negative and positive emotions (Montero-Marin et al., 2015). Therefore, we hypothesize that dispositional equanimity mediates the relationship between mindfulness and teacher burnout among early childhood educators.

### 1.3 The moderating role of empathy

Does maintaining emotional calmness alone lead to less burnout? We propose that an early childhood educator’s capacity for emotional calmness can arise from one of two scenarios: either due to adequate job competence or a reduction in emotional investment in their work. While reduced emotional investment might result in a calmer disposition, this is not the intended or desirable outcome. Given these concerns, we introduce the variable of empathy and discuss its relationship with dispositional equanimity and burnout.

Empathy is defined as the capacity to understand and share the emotional states of others (Cohen and Strayer, 1996). In educational relationships, empathy is a cognitive ability in teaching, encompassing the capacity to understand and share the emotional states of others. It serves as a fundamental process for establishing positive personal interactions. Typically, teachers with optimal levels of empathy tend to engage in inclusive practices within the classroom. Inclusivity entails ensuring that all students experience a sense of belonging, feeling valued, safe, and welcomed. This approach also contributes to the optimal development of students’ individualities (Vaquier et al., 2020). Children in the early childhood stage have limited cognitive development and are emotionally sensitive, relying on the guidance and care provided by early childhood educators in various aspects of their daily lives. Additionally, children of this age have difficulty expressing specific thoughts and complete emotions through language, so the empathetic abilities of early childhood educators can influence the emotional understanding between teachers and students. Over the course of their careers, early childhood educators encounter a myriad of emotional and educational caregiving scenarios. Due to the high emotional demands of early education environments, empathy is often considered an essential trait and skill for teaching and caring for children (Huang et al., 2020).

Previous research has found a significant negative correlation between empathy and burnout among early childhood educators (Yang et al., 2018). Studies on the relationship between personality traits and empathy have found that emotional stability is a strong and significant predictor of empathy. There is a strong positive correlation between empathy and emotional stability. Individuals with stable emotions are more likely to empathize, have better understanding and affinity, and those with a gentle and self-controlled personality are more likely to understand others objectively (Doktorová et al., 2019). This suggests that early childhood educators with high empathy abilities can better fulfill job requirements and maintain a calm mindset. Therefore, we hypothesize that empathy plays a moderating role in the relationship between dispositional equanimity and burnout among early childhood educators.

### 1.4 Research hypotheses

**Figure 1:**
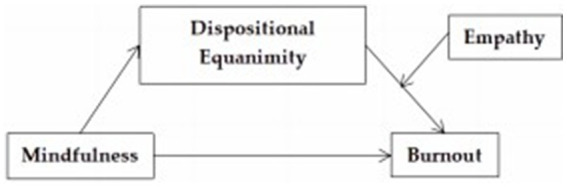
Hypothesized model illustrating moderated mediation (*n* = 1980).

In summary, the present study investigates the synergistic influences of mindfulness, dispositional equanimity, and empathy on burnout among early childhood educators, as illustrated in Figure 1. Using early childhood educators as participants, a moderated mediation model is constructed to explore the mediating role of dispositional equanimity and the moderating role of empathy in the relationship between dispositional equanimity and burnout. We posit the following three hypotheses: (1) There is a significant negative correlation between mindfulness and teacher burnout among early childhood educators. (2) Dispositional equanimity mediates the relationship between mindfulness and teacher burnout among early childhood educators. (3) Empathy moderates the relationship between dispositional equanimity and teacher burnout among early childhood educators.

## 2 Materials and methods

### 2.1 Sample and data collection

This study surveyed early childhood educators across multiple Chinese regions, including Beijing, Shanghai, Anhui, and Guangdong. All participating educators were required to complete four tests: the Empathy Scale (ME), the Maslach Burnout Inventory-Educators Survey (MBI-ES), the Mindful Attention Awareness Scale (MAAS), and the Preschool Teachers Dispositional Equanimity Questionnaire (PTDEQ). Data were collected via online questionnaires, garnering 2,039 responses. After applying the screening criteria, a total of 1,980 valid questionnaires were included, resulting in an effective response rate of 97.1% (as shown in Table 1). The screening criteria for the questionnaires in this study were as follows: 1. Correctly answered the control question (e.g., “What is 28 + 5?”); 2. Age of at least 18 years old. Anonymous questionnaires were used to ensure candid responses. Among the valid questionnaires, due to the nature of the profession, there were only 31 male participants, accounting for 1.6% of the total. The predominant age group among respondents was 22–28, comprising 39.8% of the total, followed by the 32–38 age range at 23.8%. Regarding educational attainment, 62.1% of respondents held at least a bachelor’s degree, while 37.9% had a college degree or lower. Concerning teaching experience, 50.4% had up to 5 years, 25.3% had 6–10 years, and 24.3% had more than 10 years.

**Table 1:** Demographic characteristics and related variables of study participants (*n* = 1,980).

Measure		Mean ± SD/n (%)
Gender		
	Male	31 (1.6)
	Female	1949 (98.4)
Age		31.2 ± 8.4
Length of teaching		
	5 years and below	998 (50.4)
	6–10 years	502 (25.3)
	11–21 years	192 (9.7)
	16–20 years	70 (3.5)
	Over 21 years	218 (11)
Province		
	Anhui	1,386 (70)
	Beijing	52 (2.6)
	Guangdong	85 (4.3)
	Shanghai	454 (22.9)
	Other	3 (0.1)
Highest education level		
	Bachelor’s degree or above	1,229 (62.1)
	Junior college or below	751 (37.9)
School type		
	Urban public kindergarten	823 (41.6)
	Urban private kindergarten	287 (14.5)
	Township Public Kindergarten	799 (40.4)
	Township Private Kindergarten	71 (3.6)
Teaching grade		
	Senior	718 (36.3)
	Junior	673 (34)
	Middle	589 (29.7)

### 2.2 Measurement tools

#### 2.2.1 Mindful attention awareness

When used in mainland China, this psychological measurement tool has demonstrated excellent psychometric properties (Deng et al., 2012). The Chinese adaptation of the MAAS is a unidimensional scale comprised of 15 items, such as “Sometimes I am unaware of my feelings until later on.” These items are related to various aspects of cognitive, emotional, and physiological experiences in daily life. The items are rated on a 6-point scale, ranging from 1 (“almost always”) to 6 (“almost never”). All items are positively scored, with higher scores indicating higher levels of present-moment mindful attention awareness. In this study, the Chinese version of the MAAS had a McDonald Omega coefficient of 0.937,which exceeds the acceptable threshold of 0.7 (Trizano-Hermosilla and Alvarado, 2016).

#### 2.2.2 Teacher burnout

The Maslach Burnout Inventory-Educators Survey (MBI-ES) was used in this study (Aboagye et al., 2018). This inventory consists of 22 items that cover three dimensions: emotional exhaustion, depersonalization, and reduced personal accomplishment. The items are rated on a 5-point Likert scale, ranging from 1 (“never”) to 5 (“always”). An example of the questionnaire is: “After a day’s work, I feel exhausted.” The items in the reduced personal accomplishment dimension are reverse scored, while the items in the emotional exhaustion and depersonalization dimensions are positively scored. A higher total score indicates a higher level of teacher burnout. In this study, the MBI-ES demonstrated good reliability, with a McDonald Omega coefficient of 0.733.

#### 2.2.3 Dispositional equanimity of preschool teachers

The Preschool Teacher Dispositional Equanimity Questionnaire (PTDEQ) developed by He and others was used in this study (He, 2020). This questionnaire consists of 15 items that cover three dimensions: dispositional calmness, dispositional gentleness, and dispositional composure (He, 2020). The items are rated on a 6-point Likert scale, ranging from 1 (“completely disagree”) to 6 (“completely agree”). An example of questionnaire is: "Overall, my emotions are very stable.” All items are positively scored, and a higher total score indicates a higher level of dispositional equanimity. In this study, the questionnaire demonstrated good reliability, with a McDonald Omega coefficient of 0.948.

#### 2.2.4 Empathy

The empathy dimension of the Measure of Empathy and Sympathy (MES) questionnaire, translated by Wang Yang, Wen Zhonglin, and others, was used in this study (Wang et al., 2017b). This dimension of the questionnaire has good validity and reliability among early childhood educators in China. It includes cognitive empathy (items 1–4) and emotional empathy (items 5–8). The items are rated on a 5-point scale, ranging from 1 (“completely disagree”) to 5 (“completely agree”). An example of the questionnaire is: “Even if others do not tell me their inner feelings, I often tend to understand them.” All items are positively scored, and higher scores indicate stronger empathy abilities. The total score on this dimension ranges from 12 to 60. In this study, the empathy scale demonstrated good reliability, with a McDonald Omega coefficient of 0.837.

## 3 Data analysis

To enhance data quality and reliability, we excluded responses from participants who incorrectly answered the control question. Prior to model entry, all variables underwent z-score transformation to standardize effect sizes and mitigate multicollinearity, as recommended by Allan and Duffy (2014). Furthermore, we examined the skewness and kurtosis of all the data participating in the subsequent model testing and found that they all met the criteria of an absolute kurtosis value less than 10 and an absolute skewness value less than 3 (Kline, 2023). Therefore, we can accept that the original data of the four variables are basically normally distributed.

We used the SPSS macro PROCESS developed by Preacher and Hayes for moderated mediation analysis. Gender, age, teaching experience, highest education level, school type, and teaching grade were included as covariates in the analysis. Initially, we established a regression model controlling for empathy and sympathy to examine the impact of dispositional equanimity on teacher burnout. Subsequently, bivariate Pearson correlations were used to examine the basic associations between variables, and demographic variables were included as covariates in subsequent models. Confidence intervals (CIs) were used with a 95% level. In the correlation analysis, *p*-values less than 0.05 were considered statistically significant. Indirect effects were considered statistically significant at the 0.05 level if the 95% CIs did not include zero (Preacher and Hayes, 2008; Hayes, 2017).

## 4 Results

### 4.1 Descriptive statistics and correlation analysis

The results of descriptive statistics and correlation analysis are presented in the Table 2. We found that teacher burnout was significantly negatively correlated with dispositional equanimity, empathy, and mindfulness. Additionally, there were significant positive correlations between mindfulness and dispositional equanimity, as well as between empathy.

**Table 2:** Descriptive statistics and correlations.

Variables	1	2	3	4	M	SD
1.Burnout	–				48.17	12.566
2.Dispositional equanimity	−0.601^**^	–			77.45	11.235
3.Mindfulnes	−0.680^**^	0.551^**^	–		73.97	12.334
4.Empathy	−0.151^**^	0.212^**^	0.156^**^	–	46.62	6.445

*p < 0.05; **p < 0.01.

### 4.2 Impact of mindfulness on teacher burnout

The impact of dispositional equanimity on teacher burnout is shown in Table 3. After controlling for empathy, dispositional equanimity can directly and negatively predict the level of teacher burnout in early childhood educators.

**Table 3:** The impact of mindfulness level of preschool teachers on occupational burnout.

	B	SE	*t*	*p*
(Constant)	4.52	0.057	78.99	<0.001
Mindfulness	−0.504	0.012	−41.291	<0.001

### 4.3 Mediation analysis

The mediation effect of dispositional equanimity is presented in Table 4. In the absence of dispositional equanimity, mindfulness had a significant total effect on teacher burnout (*β* = −0.66, *t* = −39.98, *p* < 0.001). Additionally, mindfulness had a positive effect on dispositional equanimity (*β* = 0.54, *t* = 28.51, *p* < 0.001). When dispositional equanimity was included as a mediating factor, the effect of mindfulness on teacher burnout remained significant (*β* = −0.49, *t* = −26.72, *p* < 0.001), and dispositional equanimity continued to predict teacher burnout (*β* = −0.32, *t* = −17.39, *p* < 0.001). Data analysis indicated that dispositional equanimity played an important role in explaining the association between mindfulness and teacher burnout (indirect effect = −0.17, 95% CI = -0.21--0.14), as shown in Table 5.

**Table 4:** Mediation effect test of pacification tendency.

	Model 1 (DV: burnout)		Model 2 (DV: dispositional equanimity)		Model 3 (DV: burnout)	
	*β*	*t*	*β*	*t*	*β*	*t*
Age	−0.018	−5.428^***^	0.003	0.891	−0.017	−5.478^***^
Length of teaching	0.067	3.124^**^	0.030	1.207	0.077	3.825^***^
Highest education level	0.123	3.561^***^	−0.111	−2.819^**^	0.087	2.715^**^
School type	−0.010	−0.601	−0.007	−0.353	−0.012	−0.783
Teaching grade	0.021	1.061	−0.012	−0.534	0.017	0.930
Mindfulness	−0.662	−39.977^***^	0.542	28.512^***^	−0.490	−26.727^***^
Dispositional equanimity					−0.318	−17.389^***^
*R* ^2^	0.476		0.310		0.546	
*F*	298.514^***^		147.707^***^		338.148^***^	

*p < 0.05; **p < 0.01; ***p < 0.001.

**Table 5:** Mediating effect, direct effect, and total effect decomposition.

	B	Boot SE	Boot LLCI	Boot ULCI	%
Total effect	−0.662	0.017	−0.695	−0.630	
Direct effect	−0.490	0.018	−0.526	−0.454	73.98%
The mediating effect of empathy	−0.172	0.018	−0.210	−0.137	26.02%

### 4.4 Moderated mediation analysis

The results of the moderation analysis of empathy are presented in Table 6. After including empathy in the model, the interaction between empathy and dispositional equanimity was found to have a significant predictive effect on teacher burnout (*β* = −0.05, *t* = −3.77, *p* < 0.001).

**Table 6:** Moderated mediation model testing.

	DV			
	Model 1 (DV: dispositional equanimity)		Model 2 (DV: burnout)	
	B	*t*	B	*t*
Age	0.003	0.891	−0.017	−5.514^***^
Length of teaching	0.030	1.207	0.077	3.851^***^
highest education level	−0.111^***^	−2.819	0.088	2.732^**^
School type	−0.007	−0.353	−0.012	−0.745
Teaching grade	−0.012	−0.534	0.017	0.945
Mindfulness	0.542^***^	28.512	−0.483	−26.305^***^
Dispositional equanimity			−0.335	−17.519^***^
Empathy			−0.001	−0.064
Dispositional equanimity × empathy			−0.052	−3.765^***^
R	0.557		0.741	
R^2^	0.310		0.549	
F	147.707		266.308	
Conditional indirect effect analysis at values of the empathy	B	BootSE	BootLLCI	BootULCI
1(M − 1SD)	−0.153	0.020	−0.197	−0.116
0(M)	−0.181	0.018	−0.218	−0.149
−1(M + 1SD)	−0.210	0.022	−0.255	−0.168

^*^p < 0.05; ^**^p < 0.01; ^***^p < 0.001.

**Figure 2:**
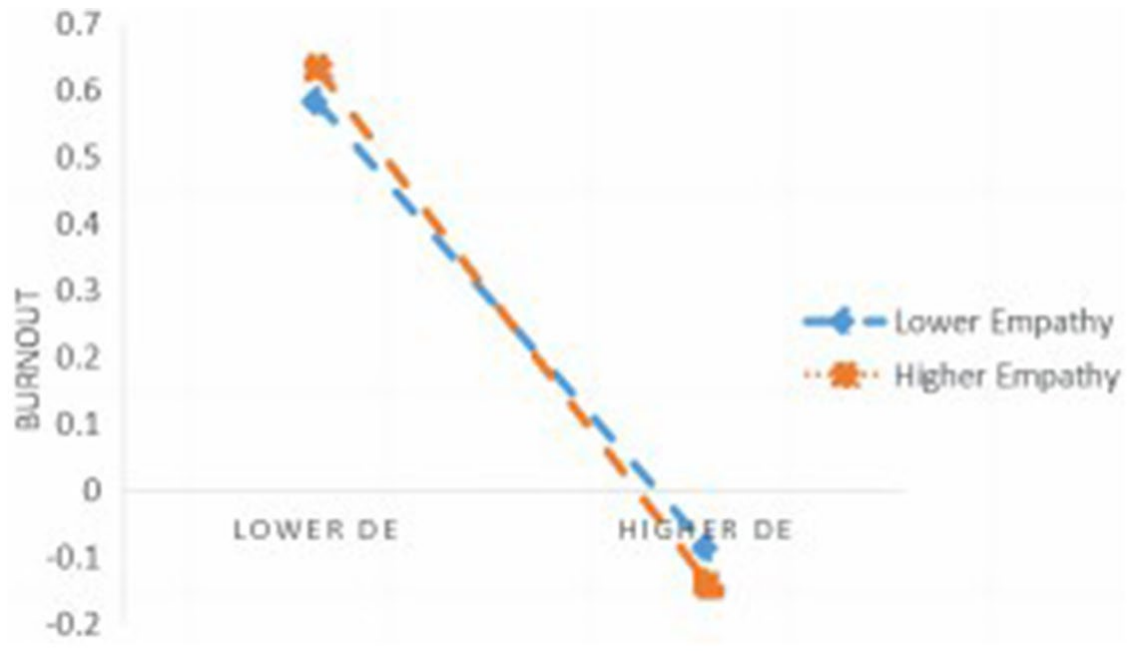
The moderating role of empathy in the relationship between dispositional equanimity (DE) and burnout.

The data suggest that empathy serves as a moderator in the relationship among mindfulness, dispositional equanimity, and teacher burnout. Further simple slope analysis (refer to Figure 2) indicates that among individuals with low empathy (M-1SD), an increase in dispositional equanimity levels correlates with a significant reduction in teacher burnout. For individuals with high empathy (M + 1SD), as dispositional equanimity levels increased, there was a significant decreasing trend in teacher burnout, and the reduction in burnout was larger compared to individuals with low empathy. Therefore, the predictive effect of dispositional equanimity on teacher burnout was strengthened with increasing levels of empathy. Thus, the negative effect of dispositional equanimity on teacher burnout in early childhood educators is enhanced with higher levels of empathy.

## 5 Discussion

This study examined the relationship between mindfulness and burnout among Preschool Teachers in China, as well as the mediating role of Dispositional Equanimity and the moderating role of empathy. The findings revealed a negative correlation between mindfulness and burnout among Preschool Teachers. Dispositional Equanimity partially mediated the relationship between mindfulness and burnout, while empathy moderated the relationship between Dispositional Equanimity and burnout.

### 5.1 The relationship between mindfulness and burnout

The results of this study support Hypothesis 1, indicating that mindfulness among Preschool Teachers significantly negatively predicts their burnout levels. This aligns with previous research (Yang et al., 2017), which suggests that mindfulness can help alleviate symptoms of work-related stress. However, existing studies have not fully elucidated how mindfulness is associated with work engagement (Leroy et al., 2013). Although the exact mechanism of how mindfulness reduces burnout remains unclear, various researchers have attempted to address this question from different perspectives. Some researchers have focused on the behavioral changes brought by mindfulness, finding that it helps individuals manage workplace stress sources by focusing on what is happening, which aids in maintaining concentration and overcoming challenges (Taylor and Millear, 2016). Others have approached the issue from the perspective of mindfulness altering individual cognition, arguing that mindfulness facilitates the perception and regulation of emotions, enabling individuals to recognize stress experiences in the workplace, accept stressful states, and adopt appropriate self-regulation strategies, thereby reducing occupational stress and burnout (Mesmer-Magnus et al., 2017). Notably, previous studies have proposed that mindfulness training can promote inner peace (Nhat and Fox, 2000; Williams and Penman, 2011; Liu et al., 2015), yet there is a lack of research directly linking mindfulness, inner peace, and burnout. Although some studies have suggested that mindfulness reduces burnout by promoting inner peace, these studies have not empirically examined inner peace as a personality trait (Moody et al., 2013; Hammer, 2021).

### 5.2 The mediating role of dispositional equanimity

By examining the relationship between mindfulness and burnout through the mediating role of dispositional equanimity, a personality trait with Chinese cultural background, this study addresses a gap in the literature (Leroy et al., 2013). The findings confirm that dispositional equanimity is a significant factor influencing burnout levels among Chinese preschool teachers and establishes its mediating role between mindfulness and burnout. Specifically, preschool teachers with higher levels of mindfulness tend to possess a higher degree of dispositional equanimity, which helps alleviate their burnout, thereby supporting Hypothesis 2. Previous research has shown that many kindergarten teachers in China are contract employees, facing high levels of temporariness and uncertainty in their work, with no guarantee of lifelong employment (Wei et al., 2021). This perceived threat to important economic and psychosocial resources can contribute to feelings of job insecurity among preschool teachers (Blackmore, 2011). Job insecurity, defined as a subjective stressor, can have negative impacts on individual work performance, physical health, and mental health (Sverke and Hellgren, 2002). Persistent job insecurity can lead to repeated negative emotional and stress-related reactions, resulting in burnout (De Witte et al., 2015). Additionally, as the duration of job insecurity increases, individuals may experience damage to their emotional stability, leading to increased neuroticism overall (Wu et al., 2020). Previous longitudinal studies have shown that individuals with higher levels of neuroticism tend to have difficulty managing negative thoughts and emotions, react strongly to recurrent stressors, and are thus more prone to burnout (Fornes-Vives et al., 2019).

Dispositional equanimity, as a significant and pervasive psychological experience, not only manifests as a state-like emotional change but also as a stable tendency for “second-order regulation” of stimulus responses. It encompasses cognitive and emotional dimensions, meaning that individuals with dispositional equanimity approach problems rationally and maintain emotional calm (He, 2020). These characteristics contrast sharply with the emotional instability, irrationality, and hostility associated with neuroticism (Yanhua and Miner, 2006). Empirical studies have also shown a significant negative correlation between dispositional equanimity and neuroticism (He, 2020). Therefore, we believe that preschool teachers with a high level of mindfulness possess the trait of dispositional equanimity, which enables them to demonstrate greater inner tranquility in their work. Additionally, they are able to face work challenges with more gentle interaction styles and rational decision-making abilities. This capability further allows them to cope with work demands and life pressures from a positive emotional perspective, reducing their perception of negative emotions in the workplace, and subsequently mitigating the level of burnout.

### 5.3 The moderating role of empathy

The relationship between empathy and dispositional equanimity deserves attention as well. When individuals encounter challenges at work, they may maintain inner tranquility by approaching problems in a positive and rational manner, or by reducing their involvement in work. Obviously, the latter is undesirable. Therefore, we explored the role of empathy in the relationship between dispositional equanimity and burnout. According to our research findings, preschool teachers with higher empathy demonstrate a stronger negative effect on the impact of dispositional equanimity on burnout compared to those with lower empathy. This suggests that reducing burnout by decreasing involvement in work is not effective. Drawing on previous studies, we speculate that this is due to the unique nature of the teaching profession: preschool teachers with higher empathy tend to have a greater sense of adequacy in their work, enabling them to face events in work and life with a more calm mindset (Peck et al., 2015). This helps reduce psychological exhaustion and, consequently, burnout levels.

Specifically, numerous studies have shown that empathy is associated with more successful interpersonal conflict management strategies, such as actively solving problems (de Wied et al., 2007). Furthermore, empathy, as the ability to perceive and understand others’ emotions (Goldstein and Michaels, 2021), manifests in preschool teachers as the ability to perceive and understand children’s emotions. Therefore, we believe that preschool teachers with high empathy can more sensitively detect children’s emotional needs and respond accordingly, increasing their level of engagement in work. Empathy is highly positively correlated with dispositional equanimity and mindfulness, indicating that preschool teachers with higher empathy are also more inclined to adopt a more rational cognitive mode to cope with stressful events in work and life. Therefore, we consider the empathy of preschool teachers as a protective factor for work (Demerouti et al., 2001).

In summary, we believe that mindfulness, dispositional equanimity, and empathy can all serve as work resources to protect preschool teachers from the harms of burnout. At the same time, our findings suggest future research directions: the impact of mindfulness interventions on burnout can be achieved through changes in personality traits. Although personality traits are highly stable factors, studies have found that interventions such as psychotherapy may lead to changes in personality (McCrae, 1991).

### 5.4 Limitations

This study has several limitations to consider. Firstly, the factors that influence job burnout are multifaceted, and current research mainly focuses solely on individual factors or environmental factors. Future studies can explore the combined effects of both environmental and individual factors to investigate the joint impact of personal and environmental factors on job burnout. Secondly, this study is limited to Chinese participants and lacks consideration of other cultural backgrounds. Future research should explore the concept of “Preschool Teacher burnout” within the context of human cultures. Finally, this study is a cross-sectional study, lacking supporting evidence from longitudinal research data. Future research can explore the occurrence and development process of Preschool Teacher burnout from this perspective.

## 6 Conclusion and implications

This study used structural equation modeling to investigate the influence of mindfulness on burnout among preschool teachers, as well as the mediating role of dispositional equanimity and the moderating role of empathy. The findings reveal a negative correlation between mindfulness and burnout among Chinese preschool teachers. Additionally, both dispositional equanimity and empathy had significant effects on the level of burnout among preschool teachers, mediating and moderating the relationship between mindfulness and burnout. These findings highlight the importance of mindfulness and dispositional equanimity for the well-being of preschool teachers.

Furthermore, the moderated mediation model in this study has practical implications for preventing burnout among preschool teachers. First, mindfulness demonstrates a negative predictive relationship with occupational burnout in preschool educators. This suggests that mindfulness training may be used as a preventive intervention for preschool teachers at risk of developing burnout (Daya and Hearn, 2018). Research has shown that mindfulness meditation training can increase mindfulness, reduce neuroticism, and decrease burnout among female teachers in schools (Fabbro et al., 2020). Strengthening mindfulness among preschool teachers can improve their job satisfaction and positively impact the children they care for (Farewell et al., 2022). Second, our study partially explains the mechanisms through which mindfulness affects the level of burnout among preschool teachers. According to the emotion regulation model of mindfulness (Mesmer-Magnus et al., 2017), mindfulness as a protective factor helps individuals perceive and regulate their emotions, allowing them to recognize their stress experiences in the workplace, accept stress states, and employ appropriate self-regulation strategies. This cultivates internal calmness and enables preschool teachers to approach their work with rationality and gentleness (He, 2020). The internal calmness and rational behavior help preschool teachers better cope with work challenges, resulting in less emotional exhaustion and lower levels of burnout.

Finally, to address the issue of whether having sufficient job competence or reducing emotional involvement in work can lead to greater dispositional equanimity and lower levels of burnout among preschool teachers, we introduced empathy as a variable and examined its role. The results indicate that empathy enhances the negative effect of dispositional equanimity on burnout. This aligns with the conclusion of the JD-R model of burnout, which suggests that higher work-protective factors can reduce burnout levels (Demerouti et al., 2001). Given the unique nature of the teaching profession, preschool teachers face higher emotional labor requirements compared to other professions, and empathy can serve as one of the work-protective factors to meet these demands. Empathy is essential for helping and caring professions, and increasing empathy can prevent burnout resulting from work demands (Wilkinson et al., 2017). Consequently, preschool teachers can enhance their job performance and reduce burnout by improving their empathic ability, rather than engaging in less emotional labor in their work. This conclusion helps preschool teachers understand the relationship so that to reduce burnout in their work, they need to enhance their ability to cope with emotional labor demands. This suggests the importance of fostering empathy among preschool teachers and provides support for previous researchers’ assertion that empathy should be a key component of all early childhood teacher education programs (Peck et al., 2015).

In conclusion, this study contributes to the understanding of the impact of mindfulness, dispositional equanimity, and empathy on burnout among preschool teachers. The findings highlight the importance of cultivating mindfulness, promoting dispositional equanimity, and enhancing empathic abilities as potential strategies to prevent burnout and promote the well-being of preschool teachers. Future researches should further explore the effectiveness of interventions targeting mindfulness, dispositional equanimity, and empathy in reducing burnout and enhancing job satisfaction among preschool teachers.

## Data availability statement

The raw data supporting the conclusions of this article will be made available by the authors, without undue reservation.

## Ethics statement

The studies involving humans were approved by Anhui Normal University (reference number BBA190027). The studies were conducted in accordance with the local legislation and institutional requirements. The participants provided their written informed consent to participate in this study.

## Author contributions

YH: Conceptualization, Funding acquisition, Investigation, Methodology, Project administration, Resources, Supervision, Writing – original draft, Writing – review & editing. XW: Conceptualization, Data curation, Formal analysis, Methodology, Validation, Visualization, Writing – original draft, Writing – review & editing. XQW: Conceptualization, Methodology, Project administration, Supervision, Validation, Writing – original draft, Writing – review & editing. XL: Investigation, Supervision, Writing – review & editing.
